# Pretreatment predictors of adjuvant chemoradiation in patients receiving transoral robotic surgery for squamous cell carcinoma of the oropharynx: a case control study

**DOI:** 10.1186/s41199-016-0008-7

**Published:** 2016-07-04

**Authors:** Harry E. Subramanian, Henry S. Park, Andrea Barbieri, Amit Mahajan, Benjamin L. Judson, Saral Mehra, Wendell G. Yarbrough, Barbara A. Burtness, Zain A. Husain

**Affiliations:** 1 Department of Therapeutic Radiology, Yale University School of Medicine, PO Box 208040, New Haven, CT 06510 USA; 2 Department of Pathology, Yale University School of Medicine, New Haven, CT USA; 3 Department of Diagnostic Radiology, Yale University School of Medicine, New Haven, CT USA; 4 Section of Otolaryngology, Department of Surgery, Yale University School of Medicine, New Haven, CT USA; 5 Department of Medical Oncology, Yale University School of Medicine, New Haven, CT USA

**Keywords:** Head and Neck Cancer, Transoral Robotic Surgery, TORS, Oropharyngeal Cancer, Adjuvant Therapy, Extracapsular Extension, Positive Margins

## Abstract

**Background:**

The purpose of this study was to identify preoperative patient characteristics associated with the incidence of positive surgical margins or lymph node extracapsular extension (ECE), which necessitate adjuvant chemoradiation after transoral robotic surgery (TORS).

**Methods:**

We conducted a single institution retrospective study of 34 consecutive patients with primary oropharyngeal cancer who underwent TORS. All imaging was reviewed by a single neuroradiologist. Surgical margins and ECE status were determined by a single head and neck pathologist. Associations of preoperative patient characteristics with positive surgical margins and lymph node ECE were examined using univariate analysis. Independent predictors of these outcomes were determined using logistic regression.

**Results:**

Preoperatively, the majority of patients had early-stage disease (7 cT1 and 21 cT2; 10 cN0). Positive margins occurred in 4 (12 %) patients. A clinically positive lymph node was seen in 23 (68 %) patients. Neck dissection was performed in 29 (85 %) patients, among whom 19 had a pathologically positive lymph node and 15 had nodal ECE. Logistic regression showed that larger preoperative lymph node size was an independent predictor of ECE (odds ratio, 13.32 [95 % CI, 1.46–121.43]). Among the 21 patients with a clinically positive lymph node who underwent neck dissection, ECE was present more often in patients with a preoperative node size ≥ 3.0 vs. < 3.0 cm (92 % vs. 44 %, *P* = 0.046). There was no patient characteristic associated with positive margins.

**Conclusions:**

Patients with a larger preoperative lymph node appear more likely to have ECE, and thus be treated with chemoradiation after TORS, with a potentially higher rate of toxicity. Lymph node size should be taken into account when deciding upon treatment approaches. Further research is needed to validate these results.

**Electronic supplementary material:**

The online version of this article (doi:10.1186/s41199-016-0008-7) contains supplementary material, which is available to authorized users.

## Background

Minimally invasive transoral robotic surgery (TORS) has been rapidly adopted across the country for the treatment of oropharyngeal squamous cell carcinoma (OPSCC) [1]. The outcomes following TORS have been encouraging, with less morbidity than conventional surgery [2, 3]. However, the use of adjuvant therapy after TORS remains high [4]. Based on the landmark studies by the RTOG and EORTC, the addition of cisplatin chemotherapy is indicated in patients with extracapsular extension (ECE) of metastatic lymph nodes or positive surgical margins [5–7]. Although recent analysis has called into question the validity of these guidelines [8], adjuvant chemoradiation remains the standard of care for these high-risk patients, pending further clinical trials. Additionally, positive margins and nodal ECE are indications for dose escalation of radiation therapy [9].

Recent evidence shows nearly 75 % of newly diagnosed OPSCC in the United States are human papillomavirus (HPV) associated, with a more favorable prognosis, and as a result current trials investigate the de-escalation of therapy [10–12]. The potential toxicity advantages of TORS, however, may be negated when patients subsequently receive adjuvant high dose radiation with concurrent chemotherapy. Recent data suggest that trimodality therapy including TORS may result in increased toxicity compared to treatment with definitive chemoradiation [13]. The addition of chemotherapy, escalation of adjuvant radiation dose, and depth of resection have also been associated with an increased incidence of soft tissue necrosis after TORS [14].

Thus it remains important to carefully select patients for TORS. The purpose of this study is to obtain pretreatment predictors for margin positivity and ECE, to help optimize patient selection for TORS.

## Methods

### Patients and data collection

The medical records of all OPSCC patients treated at Yale-New Haven Hospital between January 1, 2010, and November 30, 2014, were retrospectively reviewed. Patients undergoing TORS for a primary OPSCC were extracted. Patients treated for recurrent disease were excluded. This study did not include other surgical modalities such as open surgery, because the incidence of positive margins may vary depending on surgical approach. All cases were discussed at a multi-disciplinary tumor board attended by neuroradiology, pathology, radiation oncology, medical oncology, and otolaryngology. Patients were selected for TORS if there was a consensus at tumor board that there was a high likelihood of achieving negative margins and if the lymph nodes did not demonstrate obvious radiographic evidence of ECE on preoperative examination and imaging.

Information including tumor location, smoking status, p16 status, preoperative tumor size, preoperative lymph node size, neck dissection, surgical margins, ECE, pathologic tumor size, pathologic lymph node size, and receipt of adjuvant therapy were obtained. Preoperative sizes were measured from patient imaging. In order to ensure consistency, all imaging scans were re-reviewed by a single board-certified neuroradiologist, who was blinded to the results of pathologic ECE and margin status. The neuroradiologist identified radiographic ECE based on the presence of irregular borders of the lymph node capsule as well as perinodal fat infiltration. Tumor size was recorded as the largest diameter measured, and lymph node size was recorded as the long-axis diameter of the largest metastatic lymph node in each patient. Surgical margins and the presence of ECE were all re-reviewed for the purposes of this study by a single board-certified head and neck pathologist. In addition, the distance from tumor to the margin was obtained, as well as the extent of ECE, and the ECE grade based on criteria established by Lewis et al. [15]. Surgical margins after TORS were considered positive when tumor cells were present at the resection border.

### Statistical analysis

We analyzed multiple preoperative characteristics of these patients as independent variables to identify possible associations to nodal ECE or positive surgical margins. All analyses were performed using SPSS version 19 (IBM SPSS, Armonk, NY). Chi-square and logistic regression analysis were used to identify predictors of positive margins and ECE. Factors associated with a *P* value < 0.10 in univariate analyses were included in multivariable analysis. The study was approved by the Yale University Institutional Review Board, and informed consent was waived.

## Results

### Patient characteristics

A total of 34 patients with primary squamous cell carcinoma of the oropharynx undergoing TORS were identified. Overall patient characteristics are summarized in Table 1. The median age was 56.5 years, 28 (82 %) patients had p16-positive tumors, and 21 (62 %) patients were current or former smokers. Preoperatively, the majority of patients had early-stage disease, with seven (21 %) T1 tumors and 21 (62 %) T2 tumors. A total of 10 (29 %) patients were staged cN0. The overall clinical staging of our patients was distributed as: two (6 %) stage I, six (18 %) stage II, 11 (33 %) stage III, and 14 (42 %) stage IVA. The overall pathologic staging of our patients was distributed as: four (12 %) stage I, five (15 %) stage II, six (18 %) stage III, and 18 (53 %) stage IVA. In total, eight (24 %) patients were upstaged while three (9 %) patients were downstaged postoperatively. The other 21 (62 %) patients remained the same stage, and two (6 %) were not evaluable due to missing staging information. The median time between pre-treatment imaging studies and surgery was 29 days. Based on measurements from imaging, the median preoperative tumor size was 2.20 cm, and the median preoperative lymph node size was 3.00 cm.

**Table 1:** Patient characteristics (*n* = 34)

Characteristic	Patients
Age (Median)	56.5
Sex	
Male	25 (74 %)
Female	9 (27 %)
Location	
Tonsil	17 (50 %)
Base of Tongue	16 (47 %)
Soft Palate	1 (3 %)
Smoking	
Smoker	21 (62 %)
Non-Smoker	13 (38 %)
p16	
Positive	28 (82 %)
Negative	3 (9 %)
Undetermined	3 (9 %)
Preoperative Tumor Size (median, *n* = 25)	2.20 cm
Postoperative Tumor Size (median, *n* = 31)	2.20 cm
Preoperative Node Size (median, *n* = 23)	3.00 cm
Postoperative Node Size (median, *n* = 19)	3.50 cm
Preoperative T Stage	
T0	3 (9 %)
T1	7 (21 %)
T2	21 (62 %)
T3	2 (6 %)
T4	0 (0 %)
Unknown	1 (3 %)
Postoperative T Stage	
T1	12 (35 %)
T2	17 (50 %)
T3	4 (12 %)
T4	0 (0 %)
Unknown	1 (3 %)
Preoperative N Stage	
N0	10 (29 %)
N1	9 (27 %)
N2	14 (41 %)
N3	0 (0 %)
Unknown	1 (3 %)
Postoperative N Stage	
N0	13 (38 %)
N1	3 (9 %)
N2	17 (50 %)
N3	1 (3 %)
Preoperative Overall Stage	
Stage I	2 (6 %)
Stage II	6 (18 %)
Stage III	11 (33 %)
Stage IVA	14 (42 %)
Unknown	1 (3 %)
Postoperative Overall Stage	
Stage I	4 (12 %)
Stage II	5 (15 %)
Stage III	6 (18 %)
Stage IVA	18 (53 %)
Unknown	1 (3 %)
Lymph Nodes	
Positive node on imaging	23 (68 %)
Neck dissection performed	29 (85 %)
Positive node on dissection	19 (56 %)
ECE present	15 (44 %)
No ECE present	14 (41 %)
Extent of ECE (median, *n* = 13)	2.00 mm
ECE Lewis Grade	
0	1 (3 %)
1	4 (12 %)
2	7 (21 %)
3	7 (21 %)
4	1 (3 %)
Surgical Margins	
Positive	4 (12 %)
Negative	30 (88 %)
Distance to negative margins (median)	3.00 mm
Adjuvant Therapy	
None	10 (29 %)
Radiation therapy alone	7 (21 %)
Chemoradiation therapy	17 (50 %)

*ECE* indicates extracapsular extension

A positive lymph node was detected on preoperative imaging in 23 (68 %) patients. The decision to classify a node as positive was based on review of imaging by a single head and neck radiologist. Neck dissection was performed in 29 (85 %) total patients, including 21 (62 %) who had at least one node detected on imaging. Nineteen (56 %) patients had at least one lymph node positive for carcinoma after surgery, and 15 (44 %) had nodal ECE. Only one patient without clinically detected nodes was found to be node positive after surgery, and this patient did not have ECE. Of the 19 (56 %) patients with at least one positive node after surgery, the median pathologic size was 3.50 cm.

Adjuvant therapy was given to 24 (71 %) patients, with seven (21 %) receiving radiation therapy alone and 17 (50 %) receiving chemoradiation. This was due to positive margins in one (3 %) patient, ECE in 13 (38 %) patients, and both indications in one (3 %) patient. Two (6 %) patients who had positive nodes detected prior to surgery but did not undergo neck dissection also received adjuvant chemoradiation. Three (9 %) patients were confirmed as deceased at times of 12, 23, and 36 months after surgery, among whom one (3 %) received no adjuvant therapy, one (3 %) received adjuvant chemoradiation, and one (3 %) received adjuvant radiation, respectively. Further survival analysis was not performed due to the small number of mortality events.

### Association between patient characteristics and positive margins

No patient characteristic was associated with positive margins (Table 2). Evaluated characteristics include smoking history, p16 status, tumor size, and the time interval from pretreatment imaging to surgery. Twenty-five patients (74 %) were evaluable for the examination of the association between preoperative tumor size and margin positivity. Nine patients were excluded due to a lack of preoperative tumor size due to the primary tumor not being visible on imaging (two patients), a lack of discrete tumor borders on imaging (three patients), or the absence of preoperative imaging prior to excisional biopsy (two patients) or tonsillectomy for presumed tonsillitis (two patients). Positive margins occurred in four patients (12 %). Logistic regression analysis did not show a significant association between preoperative tumor size and positive margins (odds ratio [OR] 1.72 [95 % CI, 0.26–11.45]; *P* = 0.58).

**Table 2:** Association between patient characteristics and surgical margins (*n* = 34)

Characteristic	Positive margins	Negative margins	*P* Value
Age (Mean)	58.0	56.8	.78
Sex			
Male	3 (12 %)	22 (88 %)	> .99
Female	1 (11 %)	8 (89 %)	
Location			
Tonsil	1 (6 %)	16 (94 %)	.34
Base of Tongue	3 (19 %)	13 (81 %)	
Smoking			
Smoker	3 (14 %)	18 (86 %)	> .99
Non-Smoker	1 (8 %)	12 (92 %)	
p16			
Positive	3 (11 %)	25 (89 %)	> .99
Negative	0 (0 %)	3 (100 %)	
Preoperative Tumor Size (Mean)	2.70 cm	2.18 cm	.59
Preoperative Tumor Size			
Median = 2.20 cm			> .99
Less than median size	0 (0 %)	12 (100 %)	
Greater than or equal to median size	1 (8 %)	12 (92 %)	
Postoperative Tumor Size (Mean)	3.40 cm	2.08 cm	.03
Postoperative Tumor Size			
Median = 2.20 cm			> .99
Less than median size	1 (7 %)	14 (93 %)	
Greater than or equal to median size	2 (13 %)	14 (88 %)	
Preoperative Node Size (Mean)	2.85 cm	2.89 cm	.96
Preoperative Node Size			
Median = 3.00 cm			> .99
Less than median size	1 (10 %)	9 (90 %)	
Greater than or equal to median size	1 (8 %)	12 (92 %)	
Postoperative Node Size (Mean)	3.45 cm	3.54 cm	.93
Postoperative Node Size			
Median = 3.50 cm			> .99
Less than median size	1 (13 %)	7 (88 %)	
Greater than or equal to median size	1 (9 %)	10 (91 %)	

### Association between patient characteristics and extracapsular extension

Association of patient characteristics with extracapsular extension in metastatic lymph nodes was assessed (Table 3). Lymph node size was the only preoperative characteristic associated with nodal ECE. Smoking history, p16 status, and the time interval from preoperative imaging to surgery were not associated with ECE. Twenty one (62 %) total patients who both had a positive lymph node detected on preoperative imaging and also underwent a neck dissection were evaluable for the examination of the association between preoperative node size and ECE. Patients with a larger preoperative node size were more likely to have ECE (mean 3.23 cm with ECE vs. 1.88 cm without ECE, *P* < 0.01). ECE was present more often in patients with a preoperative node size greater than or equal to vs. less than the median size of 3.00 cm (92 % vs. 44 %, *P =* 0.046). Logistic regression analysis showed that larger preoperative lymph node size was an independent predictor of ECE (OR 13.32 [95 % CI, 1.46–121.43]; *P* = 0.02).

**Table 3:** Association between patient characteristics and nodal extracapsular extension (ECE) (*n* = 29)

Characteristic	ECE	No ECE	*P* Value
Age (Mean)	55.5	58.4	.35
Sex			
Male	12 (55 %)	10 (46 %)	.68
Female	3 (43 %)	4 (57 %)	
Location			
Tonsil	8 (62 %)	5 (39 %)	.48
Base of Tongue	7 (47 %)	8 (53 %)	
Smoking			
Smoker	9 (53 %)	8 (47 %)	> .99
Non-Smoker	6 (50 %)	6 (50 %)	
p16			
Positive	14 (61 %)	9 (39 %)	.56
Negative	1 (33 %)	2 (67 %)	
Preoperative Tumor Size (Mean)	2.18 cm	2.13 cm	.86
Preoperative Tumor Size			
Median = 2.20 cm			> .99
Less than median size	6 (55 %)	5 (46 %)	
Greater than or equal to median size	4 (50 %)	4 (50 %)	
Postoperative Tumor Size (Mean)	1.90 cm	2.26 cm	.33
Postoperative Tumor Size			
Median = 2.20 cm			> .99
Less than median size	8 (53 %)	7 (47 %)	
Greater than or equal to median size	6 (50 %)	6 (50 %)	
Preoperative Node Size (Mean)	3.23 cm	1.88 cm	.002
Preoperative Node Size			
Median = 3.00 cm			.046
Less than median size	4 (44 %)	5 (56 %)	
Greater than or equal to median size	11 (92 %)	1 (8 %)	
Postoperative Node Size (Mean)	3.83 cm	2.38 cm	.03
Postoperative Node Size			
Median = 3.50 cm			.02
Less than median size	4 (50 %)	4 (50 %)	
Greater than or equal to median size	11 (100 %)	0 (0 %)	
Number of Positive Nodes on Imaging			
1	10 (71 %)	4 (29 %)	> .99
2 or more	5 (71 %)	2 (29 %)	
Number of Positive Nodes on Dissection			
1	5 (71 %)	2 (29 %)	.60
2 or more	10 (83 %)	2 (17 %)	
ECE on Imaging			
Positive	8 (89 %)	1 (11 %)	.01
Negative	7 (35 %)	13 (65 %)	
Node Necrosis on Imaging			
Positive	12 (86 %)	2 (14 %)	.001
Negative	3 (20 %)	12 (80 %)	

*ECE* indicates extracapsular extension

ECE was identified in nine (26 %) patients on imaging and in 15 (44 %) patients on pathology. ECE was identified on imaging in eight of the 15 patients with ECE confirmed on pathology (53 % sensitivity). ECE was excluded on imaging in 13 of the 14 patients without ECE on pathology (93 % specificity). In addition the positive and negative predictive values for the use of imaging to predict pathologic ECE were 89 and 65 % respectively.

A sensitivity analysis was performed to determine whether or not these findings would remain robust using different lymph node size thresholds (Table 4). ECE was also present more often in patients with a preoperative node size greater than or equal to vs. less than 2.00 cm (88 % vs. 20 %, *P* = 0.01) and in patients with a preoperative node size greater than or equal to vs. less than 2.50 cm (93 % vs. 29 %, *P* < 0.01).

**Table 4:** Sensitivity analysis comparing extracapsular extension (ECE) with preoperative node size (*n* = 21)

Preoperative Node Size	ECE	No ECE	*P* Value
Less than 2.0 cm	1 (20 %)	4 (80 %)	.01
Greater than or equal to 2.0 cm	14 (88 %)	2 (13 %)	
Less than 2.5 cm	2 (29 %)	5 (71 %)	.006
Greater than or equal to 2.5 cm	13 (93 %)	1 (7 %)	
Less than 3.0 cm	4 (44 %)	5 (56 %)	.046
Greater than or equal to 3.0 cm	11 (92 %)	1 (8 %)	
Less than 3.5 cm	10 (63 %)	6 (38 %)	.26
Greater than or equal to 3.5 cm	5 (100 %)	0 (0 %)	
Less than 4.0 cm	13 (68 %)	6 (32 %)	> .99
Greater than or equal to 4.0 cm	2 (100 %)	0 (0 %)	

*ECE* indicates extracapsular extension

## Discussion

In this series of OPSCC patients undergoing TORS, the rate of margin positivity was 12 % and the rate of nodal ECE was 44 %. No clinical features predicted positive margins in this single institution study of predominantly T1 and T2 cancers, but nodal size emerged as a significant predictor of ECE. No other clinical features were predictive of ECE. These results appear consistent with those reported from a study of the National Cancer Database (NCDB) of patients receiving TORS for oropharyngeal cancer. In that series, the rate of positive margins was 20.2 % in comparison to 12 % in the current study, suggesting our institutional results compare favorably with national practice patterns. The rate of ECE in the NCDB study was 28.5 %, although in 17 % of cases the value was unknown making it difficult to compare it to the 44 % who had ECE in the current study. Also, only nodal stage, not largest nodal size was reported in that study, again making direct comparison difficult [1].

It is a key finding of our study that the previously described relationship between nodal size and ECE [16–20] holds even in a largely HPV-associated oropharyngeal carcinoma population. HPV-associated SCC often presents with thin-walled cystic lymph nodes [21]. It is hypothesized that these cystic lymph nodes carry fewer viable tumor cells than a solid lymph node of the same size, raising the question of whether lymph node size would correlate with the risk of ECE in HPV-associated oropharynx cancer, as it did in reports from two decades ago [16–20], when HPV was much less prevalent and multiple tumor sites were studied. The current study, however, demonstrates that lymph node size remains a strong predictor of ECE even in a largely HPV-associated population. In the current study, lymph nodes 3.0 cm and larger were associated with a rate of ECE of 92 %, which was statistically significantly higher than the 44 % rate of ECE seen in smaller lymph nodes.

In addition to lymph node size, another recently investigated characteristic has been the correlation between radiographic ECE on computed tomography (CT) imaging and pathologic ECE. In our study, the use of radiographic ECE to predict pathologic ECE was associated with a sensitivity, specificity, positive predictive value (PPV), and negative predictive value (NPV) of 53, 93, 89, and 65 %, respectively. These findings are fairly consistent with a recent study of 432 patients with oral cavity or laryngeal cancer, which demonstrated a sensitivity, specificity, PPV, and NPV of 43.7, 97.7, 82.6, and 87.3 %, respectively, for the use of radiographic ECE to predict pathologic ECE, though our lower NPV may be at least partially attributable to the higher prevalence of pathologic ECE in our study (44 % vs. 20 %). The authors concluded that these values are reasonable for use in clinical decision making [22]. In another study of 100 patients with head and neck cancer, the sensitivity, specificity, PPV, and NPV by two independent radiologists were (1) 49, 84, 84, and 49 %, respectively, and (2) 65, 54, 71, and 48 %, respectively [23]. The prevalence of pathologic ECE in this study was 63 %. The authors of this study concluded that radiographic ECE is not an adequate predictor of pathologic ECE due in part to a poor NPV. The criteria used to identify radiographic ECE in our study and these two studies were irregular borders of the lymph node capsule as well as perinodal fat infiltration. Although neither of these large studies was limited to oropharyngeal cancer patients and there was a variance in the prevalence of pathologic ECE between studies, the differing conclusions demonstrate the contentious nature surrounding the use of radiographic ECE as a predictor for pathologic ECE.

It should also be noted that debate exists about how the extent of ECE affects prognosis. A study of mostly p16-positive oropharyngeal carcinoma demonstrated that while the risk of recurrence was significantly higher with grade 4 extracapsular extension (defined as no residual nodal tissue or architecture, “soft tissue metastases”), this did not appear to affect disease-free or overall survival [15]. An unrelated study of larynx and hypopharynx carcinoma demonstrated that macroscopic extracapsular extension, but not microscopic extension, was associated with risk of recurrence and death [24]. Another study of 35 patients with head and neck squamous cell carcinoma of unknown primary demonstrated lower overall and cancer-specific survival in patients with ≥ 2 mm of ECE [25]. However, a separate study of 266 patients with oral tongue cancer found no difference in survival when comparing patients with ≤ 2 mm vs. > 2 mm of ECE [26]. Current clinical trials use 1 mm of ECE as a cutoff for analysis [27], and further study is needed in this area.

The findings of the current study, if confirmed in larger series, can be used to help guide patient selection for TORS, among patients with T1 and T2 tumors. TORS is a relatively new technique for operative management of oropharyngeal tumors with less morbidity and improved functional outcomes compared to open surgical approaches for similar tumors [28–31]. Nearly 75 % of newly diagnosed oropharyngeal cancers are now associated with HPV and therefore have a more favorable prognosis, leading to current research studies investigating the benefit of de-escalation of therapy for these patients [10, 11]. Clinical trials currently underway are investigating the effectiveness of reduced-dose adjuvant radiation therapy [27] as well as definitive radiation therapy [32]. In addition, the association between ECE and prognosis in patients with p16-positive oropharyngeal cancer has lately been called into question. Recent retrospective data suggest that ECE is not associated with disease recurrence or survival in these patients [33], and this in turn has cast doubt on the necessity of adjuvant chemoradiation. The question of whether adjuvant chemoradiation is more beneficial than adjuvant radiotherapy alone in patients with ECE in the setting of HPV-related disease is currently being investigated in phase III clinical trials [34, 35]. However, the results from these studies will not be available for some time. Unfortunately, the patients in the present study were treated prior to de-escalation trials for HPV-associated OPSCC being open at our institution, and so were treated using a standard of care approach.

Limitations of this study include its retrospective design and the small sample size. Another consideration is that the patients in this study underwent TORS during the first four years of robotic surgery at our institution, and greater cumulative case volume may lead to improved outcomes. For instance, national data has shown a lower rate of positive margins in high-volume institutions (defined as having more than 10 TORS cases per year) compared to low-volume institutions [1], suggesting the existence of a “learning curve.” That said, the rate of positive margins in our study was favorable to national rates, suggesting that experience was not the critical determinant of margin positivity.

## Conclusions

The preoperative lymph node size is an independent predictor of pathologic nodal extracapsular extension in patients with OPSCC who undergo TORS. Patients with lymph nodes larger than 3 cm may be more likely to have pathologic extracapsular extension necessitating adjuvant chemoradiation, which could put them at a greater risk of requiring trimodality therapy and its associated toxicity. The likelihood of requiring trimodality therapy should be taken into account along with other factors such as patient preference, surgical risk, predicted functional outcomes, and ability to receive chemotherapy, in order to make an individualized recommendation for primary TORS or definitive chemoradiation. These findings are hypothesis-generating and warrant confirmation in larger, multi-institutional datasets. Future prospective studies are needed to evaluate functional and patient-reported outcomes, and determine the optimal management of these patients.

## Abbreviations

ECE, extracapsular extension; HPV, human papillomavirus; NCDB, national cancer database; NPV, negative predictive value; OPSCC, oropharyngeal squamous cell carcinoma; PPV, positive predictive value; TORS, transoral robotic surgery

## Additional file

Additional file 1: Database of TORS patients used for analysis. (XLSX 34 kb)
